# Design of Antimicrobially Active Small Amphiphilic Peptide Dendrimers

**DOI:** 10.3390/molecules14103881

**Published:** 2009-09-29

**Authors:** Piotr Polcyn, Margarita Jurczak, Aleksandra Rajnisz, Jolanta Solecka, Zofia Urbanczyk-Lipkowska

**Affiliations:** 1 Institute of Organic Chemistry PAS, Kasprzaka Str. 44/52 01-224 Warsaw, Poland; E-Mails: polcynp@icho.edu.pl (P.P.); rita@icho.edu.pl (M.J.); 2 National Institute of Public Health – PZH, Chocimska Str. 24, 00-791 Warsaw, Poland; E-Mails: olarajnisz@yahoo.co.uk (A.R.); jsolecka@pzh.gov.pl (J.S.)

**Keywords:** dendrimeric peptides, antimicrobial, antibiotic resistance

## Abstract

Novel polyfunctional small amphiphilic peptide dendrimers characterized by incorporation of a new core compounds – *tris*-amino acids or *tetrakis-*amino alcohols that originated from a series of basic amino acids – were efficiently synthesized. These new core elements yielded molecules with multiple branching and (+5)/(+6) charge at the 1-st dendrimer generation. Dendrimers exhibited significant antimicrobial potency against Gram(+) and Gram(-) strains involving also multiresistant reference strains (*S. aureus* ATCC 43300 and *E. coli* ATCC BAA-198). In addition, high activity against fungi from the *Candida* genus was detected. More charged and more hydrophobic peptide dendrimers expressed hemolytic properties.

## 1. Introduction

The rapid emergence of multi-drug resistance against conventional antibiotics is one of the greatest challenges of modern medical science. In places like health care units, the food industry, dental or implant materials that are confronted every day with microbial invasion new microbicides and disinfectants are an urgent need. Public awareness of the slow discovery of novel antibiotics practiced by pharmaceutical companies during last decades has recently prompted a vigorous search for complementary and alternative antimicrobial medicines characterized by new structure(s) and acting on new cellular targets [1]. Nanomedicine is one of the very promising research directions in designing novel multifunctional materials that offer better therapeutic properties in many areas of medicine, including the treatment of microbial infections [2]. 

Dendrimers - synthetic macromolecules of nanoscopic dimensions built from several layers of branches located around a central core [3,4,5] are among several groups of macromolecules that are considered as promising nanopharmaceuticals with high potential. The multivalent nature of these compounds, their unambiguous composition, reliability and versatility of their synthesis, make this type of carriers well-suited in various applications for diagnostic purposes, [6] protein mimetics, [7] antiviral agents, [8,9] vaccines, [10,11] versatile prospective drugs [12,13] and gene delivery vehicles [14]. The problem of designing antimicrobial compounds emerged in the early days of dendrimer chemistry. Cationic dendrimers with terminal ammonium groups originated from PAMAM [15], PPI [16], or carbosilane [17] dendrimers have been shown to affect the integrity of negatively charged bacterial membranes resulting in high bacteriostatic and bactericidal potency. PAMAM dendrimers have been used as carriers of quinolone [18] and penicillin V [19] antibiotics with improved bioavailability or as containers for biocidal silver nanoparticles [20].

Natural antimicrobial peptides (AMPs) constitute another groups of macromolecules that are considered a good source of new strategies and new nanopharmaceuticals [21]. Their structural diversity and mechanism of action (modification of the membrane permeability), which differs from that of the conventional antibiotics, makes them an interesting alternative to classic antibiotics. The majority of natural AMPs contain a 10-50 amino acids sequence that includes several basic amino acids (Lys, Arg) and up to 60% of these with lipophilic side chains (Phe, Tyr, Ala, etc.). Such a composition provides a positive charge and enables formation of amphipatic structure(s) essential for interactions with microbial membranes. 

Intensive research on economically feasible smaller analogs has allowed the introduction of several compounds into clinical trials [1,22]. Based on these natural compounds Tam and co-workers designed lysine (Lys) dendrimers that have been used as carriers of two to eight copies of tetra- or octapeptide fragments of the antimicrobial peptide tachyplesin. The obtained dendrimers expressed high potency against broad range of microorganisms while being at the same concentration less toxic than natural precursors [23]. 

An interesting novel application of dendrimers designed to act as new microbial targets has been reviewed recently by Rojo and Deldgado [24 and references cited therein]. These authors summarized the work of several groups focused on the synthesis of dendrimers carrying multiple copies of polysaccharides or lipopolysaccharides [25] and their intended use as infection preventing anti-adhesive agents. 

According to our concept of the “non-sequential” pharmacophore [26,27] dendrimer trees can be used not only as carriers of antimicrobial fragments, but also for *de novo* design of positively charged amphiphilic molecules. This approach allowed for the successful synthesis of a novel class of low molecular mass amphiphilic dendrimeric peptides (tetra- to octapeptides) having considerable antimicrobial activity against Gram(+) and less activity against Gram(-) bacteria with structure-dependent cytotoxicity [28,29]. According to DSC studies, these cationic amphiphilic molecules based on the Lys(Lys)_2_ scaffold interact stronger with anionic dimirystoyl phosphatidyl glycerol (DMPG)-prepared model multilamelar vesicles that emulate the properties of microbial membranes than with vesicles prepared from neutral phospholipids [30].

These studies confirmed that their selectivity is shifted towards negatively charged microbial ones rather than towards neutral human cell membranes. Although it is generally acknowledged that the structural complexity of biological membranes is a source of the selective response of microorganisms towards antibiotics the last thirty years of intensive research has provided no specific rules relating structure and biological activity of natural peptides. In the case of macromolecular dendrimeric compounds the efficiency of their interactions with heterogenic negatively charged microbial membranes depends on the number and distribution of the charged and hydrophobic groups (polyvalency) and on their structural flexibility, i.e. adaptability of the dendrimer to the dynamic structure of the membrane.

Here we present the synthesis and spectroscopic data for two groups of cationic, amphiphilic peptide dendrimers which utilize novel multibranched scaffolds of amino acid origin. Use of these new building blocks as a core element yielded first generation peptide dendrimers characterized by high charge (+5 or +6) and high polyvalency.

The antimicrobial activity of these multicharged small cationic dendrimers against both Gram(+), Gram(-) bacteria and fungi from the *Candida* genus was studied and results were compared with the activity of the previously studied less charged and less branched analogs [28]. The obtained results demonstrate that the new small but highly charged, more branched amphiphilic peptide dendrimers express better properties: along with high activity against Gram(+) bacteria, they show significant activity against Gram(-) bacteria and fungi, including antibiotic resistant strains. Hemotoxicity towards human red blood cells for more hydrophobic compounds was detected, however.

## 2. Results and Discussion

### 2.1. Synthesis

**Scheme 1:**
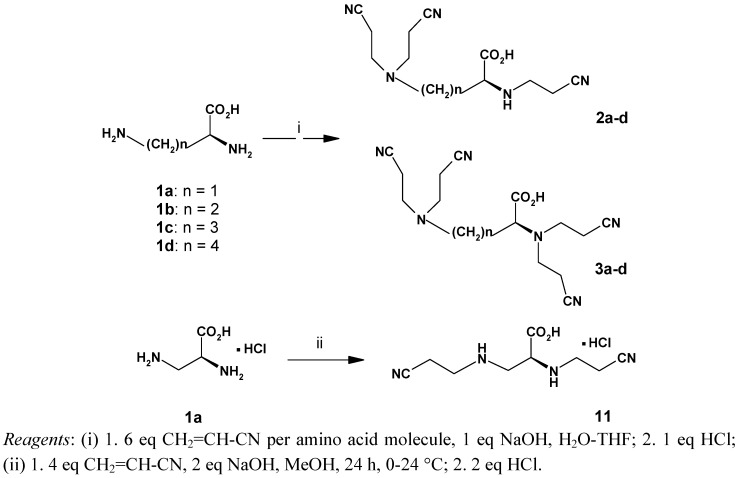
Synthesis of *N*-cyanoethylated derivatives of **1**.

Preparation of the new cores involved cyanoethylation of the respective basic amino acids (Scheme 1), followed by reduction of the nitriles to polyamines. Michael addition of acrylonitrile to polyamines (ethylene- or propylenediamine) and subsequent reduction of nitriles to yield amines is one of the classic routes in the dendrimer chemistry [31]. Condensation of acrylonitrile with several α-amino acids in aqueous solution in the presence of alkali salts, yielding mono-, di-, and tricyanoethyl derivatives was studied by McKinney [32,33].

Synthesis of a tris-*N*-cyanoethyl derivative of lysine in low yield (22.3%) was reported by Riehm and Scheraga without isolation and identification, however, of the second product – the corresponding tetrakis-*N-*cyanoethyl derivative [34]. In the case of basic amino acids, reaction with acrylonitrile gave a mixture of tris- and tetrakis-*N*-cyanoethyl derivatives with the ratio depending on the reaction conditions (Scheme 1). For example, the bis-*N-*cyanoethyl derivative **11** was initially observed as an impurity. However, when the reaction was performed at 0 °C in methanol, using four equivalents of acrylonitrile per amino group, the *N,N*-bis(cyanoethyl)-α,β-diaminopropionic acid **11** was obtained exclusively. Under the same conditions, the other three amino acids yielded tris-cyanoethyl derivatives as the major products. Most of the reduction procedures of the multiple nitrile groups involve catalytic hydrogenation with the use of the Raney-Ni or Co [35,36]. Unexpectedly, there was a pronounced difference in reactivity between tris- and tetrakis-*N*-cyanoethyl derivatives. 

**Scheme 2:**
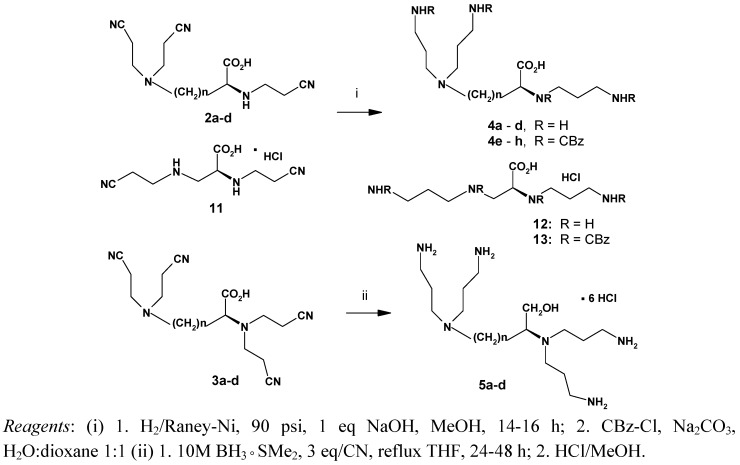
Reduction of *N*-cyanoethylated derivatives **2** and **3**.

While catalytic reduction of the tris-*N*-cyanoethyl amino acids **2** in MeOH using Raney-Ni in a Parr hydrogenation apparatus gave the respective *N*-propylamino derivatives **4** in almost quantitative yields (Scheme 2), reduction of tetrakis-*N-*cyanoethyl derivatives **3** under the above conditions left 90% of the original substrate unconverted. Finally, a family of compounds **5** was obtained using three equivalents of 10 M borane dimethylsulfide complex per nitrile group with extention of the reaction time to 24-48 h (Scheme 2) [37].

The above procedures provide a rapid access to new templates for preparation of branched peptides or other dendrimers.

**Scheme 3:**
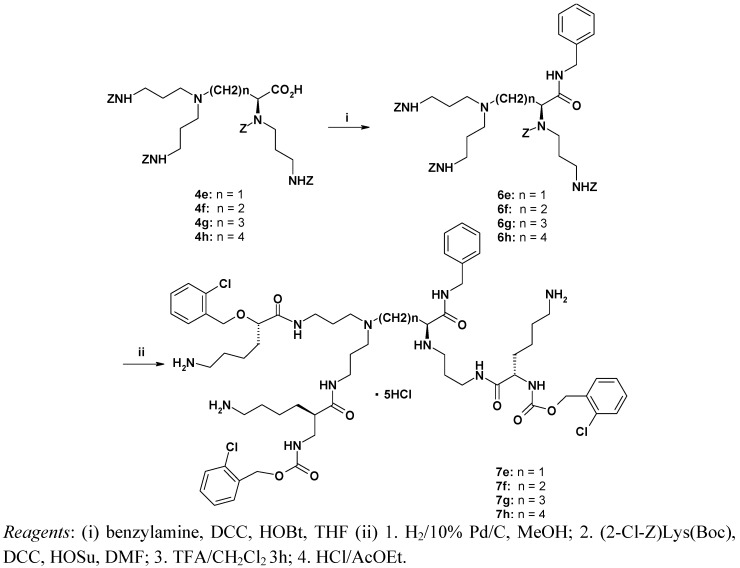
Synthesis of the dendrimers **7e-h**.

The synthesis of the lysine-functionalized first generation dendrimers **7e-h** from the *N*-protected trimeric scaffolds **4e-h** started with coupling with benzylamine, yielding the amides **6e-h**. Then the amino group deprotection was followed by coupling with (2-Cl-Z)Lys(Boc) to afford **7e-h** in 67-71% overall yield (Scheme 3). 

**Scheme 4:**
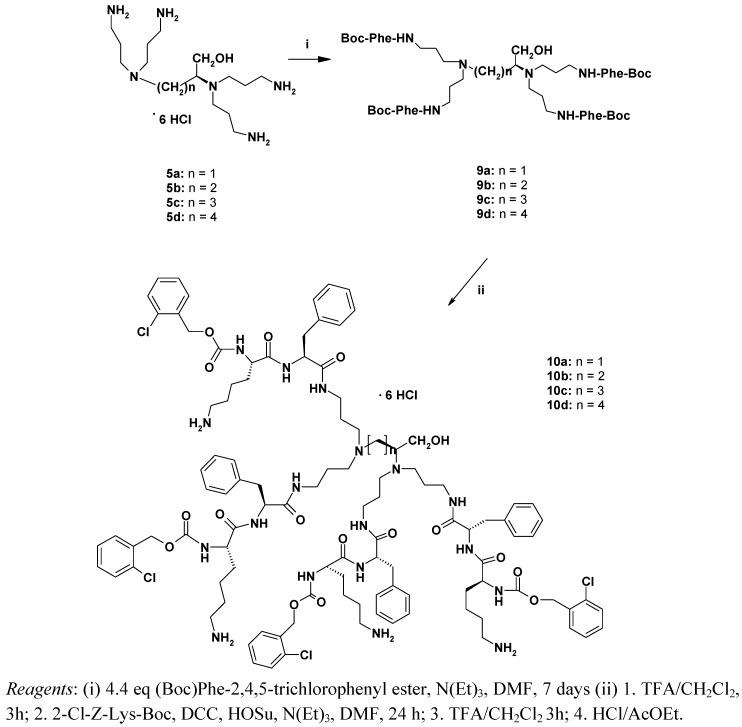
Synthesis of the dendrimers **10a-d**.

Synthesis of the tetra-functional branched compound using the hexahydrochloride of **5c** as a starting material was complicated by the fact that **5a**-**d** are practically insoluble in the commonly used organic solvents. Eventually, they were dissolved in hot DMF (50-60 °C) with addition of a large excess of triethylamine. In this case, coupling with 2,4,5-trichlorophenyl ester of (Boc)Phe was carried out for 7 days affording the compound **9a-d** in 54% yield (Scheme 4). 

### 2.1. Antimicrobial and hemolytic activities

The results of *in vitro* antimicrobial activity of the amphiphilic dendrimers **7e**-**h**, **10a**-**d** and two reference compounds **5c**-**d** assayed against two Gram(+) strains, six Gram(-) strains, and three strains from the *Candida* genus are shown in Table 1. Due to application in the synthesis 3- or 4-arm cores the families of compounds **7e**-**h** vs. **10a**-**d** are structurally different regarding the relative distribution of cationic and lipophilic groups and net charge. In the compounds **7e-h**, 3-arm cores are *N*-terminated with lysine hydrochloride residues substituted at *N^α^* with 2-chlorobenzyloxycarbonyl- residue (2-Cl-Z) and with a benzyl group located at the *C*-end (Scheme 3). In the compounds **10a-d**, all four arms of the core are elongated with phenylalanine residues (Phe) that are followed by substituted lysine residues similar to those above (Scheme 4). Within each family, compounds have the same net charge - (+5) or (+6) and differ in the number of methylene groups in the core. 

**Table 1:** Minimal inhibitory concentrations (MICs) of studied dendrimers against Gram(+), Gram(-) and fungi and two reference compounds **5c** and **5d** (μM). ^a^

Compound	7e	7f	7g	7h	10a	10b	10c	10d	5c	5d
Scaffold/charge ^b^	DAP^c^	DAB ^d^	Orn	Lys	DAP	DAB	Orn	Lys	Orn	Lys
	(+)5	(+)5	(+)5	(+)5	(+)6	(+)6	(+)6	(+)6	(+)6	(+)6
*S. aureus*	2.8	11.7	10.9	21.6	1.7	1.3	27.2	3.4	113	221
ATCC 25923										
*S. aureus*	11.8	10.3	10.9	21.6	3.4	7.3	13.6	1.7	-	-
ATCC 43300										
*P. aeruginosa*	24.3	48	21.8	86	30.2	47	54	14	-	-
ATCC 27853										
*B. bronchiseptica*	11	11	2.7	5.4	3.4	3.4	6.8	1.7	-	-
ATCC 4617										
*A. baumani*	>89	>88	5.4	>86	13.8	13.8	27.3	3.4	-	-
ATCC 19606										
*K. pneumoniae*	>89	>88	87	>86	>55	>55	54	>54	-	-
ATCC 13882										
*E.coli*	11.8	48	21.8	86	7.3	7.3	27.3	6.8	>226	>221
ATCC 25922										
*E. coli*	>88	>89	10.9	43	6.9	6.9	13.6	3.4	-	-
ATCC BAA-198										
*C. albicans*	>88	>89	10.9	>86	6.8	6.9	54	6.8	-	-
ATCC 90028										
*C. krusei*	44	44	5.4	43	6.8	6.9	27.3	3.4	-	-
ATCC 6258										
*C. parapsilosis*	88	89	5.4	86	6.8	6.9	54	3.4	-	-
ATCC 22019										

^a^ MICs of the reference compounds: Penicillin G against *S. aureus* ATCC 25923 – 6.6 (μM); polymyxin B against *E. coli* ATCC 25922 and *P. auruginosa* ATCC 27853 – 0.55 (μM); amphotericin B against *C. krusei* ATCC 6258 – 1.1 μM; indolicidin against *S. aureus* ATCC 25923 – 2.1 (μM) and *E. coli* ATCC 25922 – 4.2 (μM); ^b^ charge assigned by elementary analysis from Cl content; ^c^ diaminopropionic acid core; ^d^ diaminobutyric acid core.

**Figure 1:**
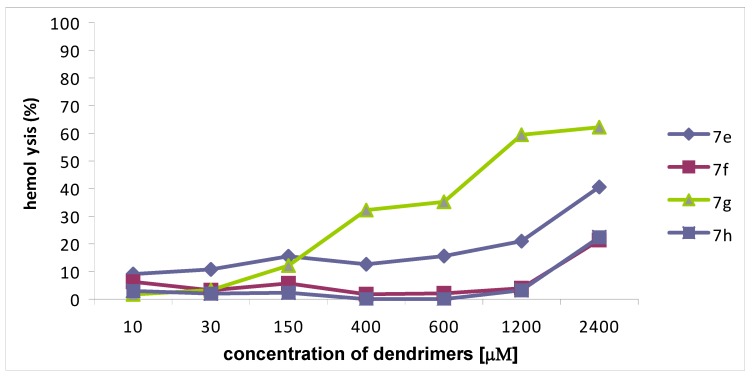
Hemolytic activity of the three-arm compounds **7e-h**.

**Figure 2:**
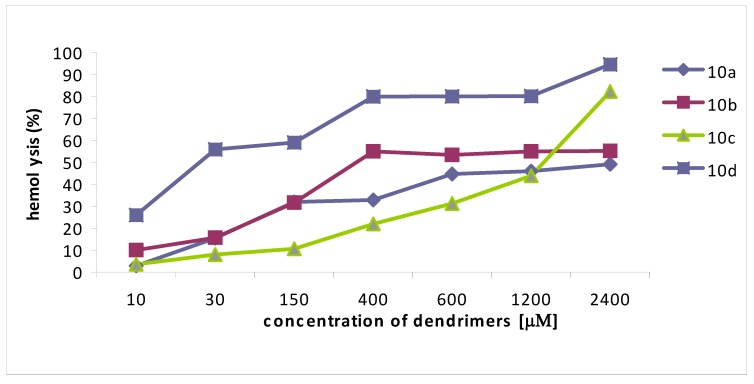
Hemolytic activity of the four-arm compounds **10a**-**d**.

Dendrimers **7e**,**f**,**h** characterized by the MIC values in the range 2.8-21.6 μM displayed less than 30% hemotoxicity up to a concentration 1,200 μM (extrapolated), whereas dendrimer **7g**, exhibited 32% toxicity at a concentration of 400 μM (Figure 1). Compounds of the second series **10a**,**b**,**c** characterized by MICs in the 1.3-54 μM range displayed 30% hemolysis at 400, 150 and 600 μM concentrations, respectively (Figure 2). Although hemotoxicity of the most potent compound **10d** characterized by the MIC values in the range 1.7-14 μM reaches 25% in the region of antimicrobial activity. From this group of compounds the best antimicrobial activity against *Candida* genus and low hemolytic effect correspond to dendrimer **7g** built around the smallest diaminopropionic acid core (DAP), and dendrimer **10a**, that presents a similar antimicrobial profile and significantly lower hemotoxicity than compound **10d**.

Selectivity of molecule-membrane interactions has been the main concern of numerous studies on biomedical applications of both multi-charged antimicrobial peptides [39,40] and dendrimers (PAMAM, PPI, etc.) [41,42]. Multiple positive charge and amphiphatic structure are proposed as a source of higher affinity of natural peptides to microbial *versus* human membranes [22]. In case of the title peptide dendrimers, the charge comes from the protonated amino groups of lysines (3 or 4) and two protonated ternary N-atoms from the core. Accumulation of positively charged groups in relatively small branched molecules should promote extended “carpet-like” conformations, facilitating multiple electrostatic interactions with microbial membranes composed mainly from acidic phospholipids (phosphatidylglycerol and cardiolipin). In contrast, outer leaflets of mammalian membranes are mainly composed from zwitterionic phospholipids (phosphatidylcholine and sphingomyelin) with only minor contribution of negatively charged species. Low impact of cationic compounds on red blood cells is regarded as an indication of a compound’s selectivity. In the present case, differences in hemolytic properties are observed within compounds belonging to the same series and even more pronounced between structurally different **7e-h** and **10a-d** series. Four-arm dendrimers **10a-d**, [(+6) charge] containing four additional hydrophobic side chains from phenylalanine residues expressed higher antimicrobial potency associated however, with enhancement of hemolytic properties. Similarly, recent data on cationic, amphiphilic polymethacrylates revealed that lower mole fraction of hydrophobic groups is one of the important factors in design of potent, non-hemolytic antimicrobial polymers [43].

The presented data show that designing more charged amphiphilic peptide dendrimers resulted in better antimicrobial properties. It appears however, that an increase of charge from (+5) to (+6) is not sufficient for significant enhancement of antimicrobial potency between **7e-h** vs. **10a-d** series and for shift in antimicrobial profile. Both, antimicrobial activity and hemolytic properties of dendrimeric peptides depend also on distribution of charged and lipophylic groups, i.e. on structural factors. Unlike many other dendrimer types, dendrimeric peptides constructed from, or terminated with basic amino acids allow to distribute on the surface independently two different types of residues – e.g. charged and lipophilic. Their number, relative sizes and orientations may be varied providing an opportunity to obtain dendrimers with good antimicrobial activity and low toxicity. 

## 3. Experimental

### 3.1. General

All solvents and reactants were of analytical grade and were used without further purification. Mass spectra were recorded with a Mariner ESI time-of-flight mass spectrometer (PerSeptive Biosystems) for the samples prepared in MeOH. Proton and carbon NMR spectra were recorded using a Bruker Avance spectrometer at 500 or 400 MHz, respectively, using deuterated solvents and TMS as an internal standard. Chemical shifts are reported as δ values in parts per million and coupling constants are given in hertz. The optical rotations were measured with JASCO J-1020 digital polarimeter. Melting points were recorded on a Köfler hot-stage apparatus and are uncorrected. Thin layer chromatography (TLC) was performed on aluminum sheets with silica gel 60 F_254_ from Merck. Column chromatography (CC) was carried out using silica gel (230-400 mesh) from Merck or Sephadex LH20. The TLC spots were visualized by treatment with 1% alcoholic solutions of ninhydrin and heating.

### 3.2. Antifungal susceptibility testing

*Yeasts:* *Candida albicans* ATCC 90028, *Candida parapsilosis* ATCC 22019 and *Candida krusei* ATCC 6258 were cultivated onto Sabouraud dextrose agar (Difco) during 24 hrs at 35 °C. 

*Medium:* RPMI – 1640 (Sigma) supplemented with 0.2% (wt/vol) glucose, 0.3 g/L L-glutamine, 0.0053 g/L phenol red, buffered with morpholinopropanesulfonic acid (MOPS; Sigma) at a final concentration of 0.165 M and adjusted to pH 7.0 was prepared. 

*Reference Method:* The broth microdilution susceptibility test was performed as described in Clinical and Laboratory Standards Institute (CLSI) reference method M27 – A2 (1) [44]. A series of the twofold dendrimers and amphotericin B dilutions in DMSO were diluted 1:49 with RPMI. Aliquots (100 µL0 were dispensed into microdilution sterile plates (Mar-Four). Then, yeast inoculum (100 µL) containing 1 × 10^3^ to 5 × 10^3^ CFU/mL was added to each well. The final concentration of dendrimers ranged from 128 to 2 µg/mL [or 105 to 1.6 μM for an average molecular mass for **7** series (1,213) and 61 to 0.9 μM for an average molecular mass for **10** series (2,103)], and amphotericin B from 1 to 8 µg/mL (1.1 to 8.1 μM, respectively), all in twofold dilution steps. The plates were incubated at 35 °C and read after 24 and 48 hrs. MIC’s (Minimal Inhibitory Concentration) was defined as the lowest drug concentration that reduced growth by 100%.

### 3.3. Antibacterial susceptibility testing

*Bacteria*. *Staphylococcus aureus* ATCC 25923, *Pseudomonas aeruginosa* ATCC 27853, *Escherichia c*oli ATCC 25922 and *Staphylococcus aureus* ATCC 43300 were cultivated on tryptone - soy agar (TSA; Oxoid). *Klebsiella pneumoniae*ATCC 13882, *Bordetella bronchiseptica* ATCC 4617, *Acinetobacter baumannii* ATCC 19606 were cultivated onto nutrient agar (Difco). *Escherichia coli* ATCC BAA-198 was cultivated on TSA with ceftazidime (10 µg/mL or 15.7 μM; Sigma). All strains were incubated during 24 hrs at 37 °C.

*Medium*. Mueller – Hinton Broth (Oxoid) was supplemented with cations: 12.5 mg Mg ^++^/L and25 mg Ca^++^/L. The pH of the medium after sterilization is between 7.2 and 7.4 [cation–adjusted Mueller – Hinton Broth” (CAMHB)].

*Reference method.* Broth microdilution susceptibility test was performed as described in Committee Laboratory Standards (CLSI) reference method M7-A7 (2) [45]. A series of the twofold dendrimers dilutions in DMSO and twofold polymyxin B and penicillin G dilutions in CAMHB were diluted 1: 94 with CAMHB. Aliquots (95 µL) were dispensed into microdilution sterile plates (Mar-Four). Then, bacteria inoculum (5 µL) containing 5 × 10^4^ CFU/mL were added. The final concentration of dendrimers ranged from 128 to 2 µg/mL [or 105 to 1.6 μM for an average molecular mass for **7** series (1213) and 61 to 0.9 μM for an average molecular mass for **10** series(2,103)], polymyxin B and penicillin G from 8 – 0.15 µg /mL (or 5.8 to 0.1 μM and 21.5 to 0.4 μM, respectively for polymyxin B and penicillin G), all in twofold dilution steps. The plates were incubated at 35 °C and read after 18 or 24 hours depending on bacterial strain. MICs (Minimal Inhibitory Concentration) was defined as the lowest drug concentration that reduced growth by 100%.

### 3.4. Hemolysis assay

Dendrimer induced hemolysis was observed as previously reported [18]. Briefly, serum free human red blood cells obtained from the Institute of Hematology and Transfusion Medicine in Warsaw, were suspended in phosphate buffered saline (PBS, pH 7.4). Prepared suspension of 1% hematocrit was incubated with serial concentration of dendrimers for 30 min at 23 °C. After centrifugation (1,000 rpm, 5 min) the absorbance of supernatant was measured at 540 nm (Jasco 630, Japan). A value of 100% hemolysis was determined by incubation of erythrocytes with double-distilled water (30 min at 23^o^). In a control experiment, cells were incubated in buffer without peptide and absorbance at 540 nm value was used as a blank.


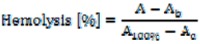


A - absorbance of the samples incubated with dendrimers; A_b_ – absorbance of the blank samples; A_100%_ - absorbance of the reference; A_c_ - absorbance of red blood cells in PBS, hematocrit 1%.

### 3.5. Synthesis

#### 3.5.1. Substrate preparation

The monohydrochlorides of L-α,β-diaminopropionic acid (**1a**) and L-α,γ-diaminobutyric acid (**1b**) were obtained according to the Rao protocol as white crystals, with the correct melting points, elemental analysis and optical rotation values: α[D]^28,5°C^ = +25 ± 1º and +24 ± 1º (C=2 in 1M HCl) for **1a** and **1b**, respectively, corresponding to the literature data [46].

To a stirred aqueous solution containing 0.1 mol of a basic amino acid and 0.1 mol of NaOH, an excess of acrylonitrile (0.6 mol) and THF (30-40 mL) was added. The mixture was stirred for 48 h at room temperature and then refluxed for 3 h. To a cold reaction mixture, 0.1 mol of conc. HCl was added and left in refrigerator overnight. The crystalline product was separated and washed with several portions of cold MeOH-H_2_O (1:1). The washings were collected and evaporated to dryness. Then acetone (150 mL) was added to the oily residue and filtered. Two crops of precipitate were combined and recrystallized from hot water or MeOH-H_2_O (7:3) giving the tris-*N*-cyanoethylated amino acids **2a**-**d** as white crystals in 49.5-69.2% yield.

The acetone filtrate was evaporated to dryness and purified by flash chromatography, eluting with an 8:2 mixture of ethyl acetate and hexane plus 3% of methanol, yielding the tetrakis-*N*-cyanoethylated derivatives **3a**-**d** as amber-colored gums (except N^α^,N^β^-tetrakis(cyanoethyl)-L-α,β-diaminopropionic acid, which slowly crystallized to give a white solid) in 21.1-28.6% yield. Both the yields and the **2**:**3** ratio depend on the reaction temperature. The yield of **3** can reach 50% when the reaction is performed in a boiling mixture of water-methanol (1:1) overnight.

*N,N,N’-tris(cyanoethyl)-L-α,β-diaminopropionic acid* (**2a**): Yield: 13.0 g (49.5%); mp 178-180 °C (dec.); ESI MS 264 (M+1), 286 (M+23); ^1^H-NMR (D_2_O) δ 2.74 (m, 4H, βCH_2_CN), 2.97 (m, 2H, αCH_2_CN), 2.99 (dd, J = 3.96 Hz, 1H, βCH**_a_**), 3.05 [m, 4H, N_β_-(CH_2_)_2_], 3.15 (dd, J = 3.96 Hz, 1H, βCH**_b_**), 3.6 (t, *J* = 6.96 Hz, 2H, NH^α^-C***H***_2_), 3.9 (dd, *J* = 3.96 Hz, 1H, αCH); ^13^C-NMR δ 15.2 (α***C***H_2_-CN), 15.4 (β***C***H_2_-CN), 42.7 (NH^α^-CH_2_), 48.8 [N^β^-(CH_2_)_2_], 53.2 (βC), 61.1 (αC), 117.6 (αCN), 121.0 (βCN), 170.9 (COOH); Anal. Calcd for C_12_H_17_O_2_N_5_: C, 54.7; H, 6.5; N, 26.6. Found: C, 54.53; H, 6.42; N, 26.44; α[D]^26.2^^°^^C^ = +24.3 ± 1° (c = 2 in 1M HCl).

*N,N_,_N’-tris(cyanoethyl)-L-α,γ-diaminobutyric acid* (**2b**): Yield: 17.3 g (62.6%); mp 170-172 °C; ESI MS 278 (M+1), 300 (M+23); ^1^H-NMR (D_2_O) δ 2.09 (q, *J* = 6.55 Hz, 2H, βCH_2_), 2.73 (m, 6H, CH_2_CN), 2.93 [m, 4H, N_γ_-(CH_2_)_2_], 3.03 (t, *J* = 6.85 Hz, 2H, NH^α^-C***H***_2_), 3.46 (t, *J* = 6.85 Hz, 2H, γCH_2_), 3.84 (t, *J* = 6.08 Hz, 1H, αCH); ^13^C-NMR δ 15.5 (α***C***H_2_-CN), 15.5 (γ***C***H_2_-CN), 26.9 (βC), 42.7 (NH^α^-CH_2_), 48.2 [N_γ_-(CH_2_)_2_], 50.0 (γC), 62.4 (αC), 117.9 (αCN), 121.2 (γCN), 172.9 (COOH); Anal. Calcd for C_13_H_19_O_2_N_5_: C, 56.30; H, 6.90; N, 25.25. Found: C, 56.05; H, 6.80; N, 25.20; α[D]^27.8^^°C^ = +24.2 ° 1° (c = 2 in 1M HCl). 

*N,N,N’-tris(cyanoethyl)-L-ornithine* (**2c**): Yield: 18.4 g (63.3%); mp 192-194 °C; ESI MS 292 (M+1), 314 (M+23); ^1^H-NMR (D_2_O) δ 1.51, 1.92 (2m, 4H, γCH_2_, βCH_2_), 2.62 (t, *J* = 7.28 Hz, 2H, αCH_2_CN), 2.69 (t, *J* = 6.65 Hz, 4H, δCH_2_CN), 2.91 [t, *J* = 6.73 Hz, 4H, N^δ^-(CH_2_)_2_], 3.02 (t, 2H, NH^α^-C***H***_2_), 3.42 (m, 2H, δCH_2_), 3.72 (dd, *J* = 4.95 Hz, 1H, αCH); ^13^C-NMR δ 15.3 (α***C***H_2_-CN), 15.7 (δ***C***H_2_-CN), 22.0 (γC), 27.6 (βC), 42.3 (NH^α^-CH_2_), 48.5 [N^δ^-(CH_2_)_2_], 52.3 (δC), 62.9 (αC), 117.9 (αCN), 121.1 (δCN), 173.4 (COOH); Anal. Calcd for C_14_H_21_O_2_N_5_: C, 57.71; H, 7.26; N, 24.03. Found: C, 57.52; H, 7.28; N, 23.85; α[D]^27.5^^°^^C^ = +24.5 ± 1° (c = 2 in 1M HCl).

*N,N,N’-tris(cyanoethyl)-L-lysine* (**2d**): Yield: 21.1 g (69.2%); mp 214-216 °C; ESI MS 306 (M+1), 328 (M+23); ^1^H-NMR (DMSO) δ 1.30-1.38 (bm, 4H, γCH_2_, δCH_2_), 1.50-1.55 (2m, 2H, βCH_2_), 2.45 (t, *J* = 7.05 Hz, 2H, εCH_2_), 2.55 (m, 6H, CH_2_CN), 2.73 [t, *J* = 6.76 Hz, 4H, N^ε^-(CH_2_)_2_], 2.65, 2.81 (2m, 2H, NH^α^-C***H***_2_), 3.15 (t, *J* = 6.42 Hz, 1H, αCH); ^13^C-NMR δ 15.5 (ε***C***H_2_-CN), 17.7 (α***C***H_2_-CN), 22.8 (γC), 26.5 (δC), 32.0 (βC), 42.9 (NH^α^-CH_2_), 48.4 [N^ε^-(CH_2_)_2_], 52.3 (εC), 60.2 (αC), 119.7 (αCN), 119.9 (εCN), 175.2 (COOH); Anal. Calcd for C_15_H_23_O_2_N_5_: C, 58.99; H, 7.59; N, 22.9. Found: C, 58.9; H, 7.47; N, 22.89; α[D]^22^^°^^C^ = +23 ± 1° (c = 2 in 1M HCl).

*N_,_N’,N,N’-tetrakis(cyanoethyl)-L-α,β-diaminopropionic acid* (**3a**): Yield: 9.05 g (28.6%); mp 126-128 °C; ESI MS 317 (M+1), 339 (M+23); ^1^H-NMR (DMSO) δ 2.57-2.67 (bm, 9H, CH_2_CN, βCH**_a_**), 2.81-2.98 [bm, 9H, N-(CH_2_)_2_, βCH**_b_**], 3.48 (dd, *J* = 6.24 Hz, 1H, αCH), 12.64 (s, 1H, COOH); ^13^C-NMR δ 15.3, 17.4 (α***C***H_2_-CN, β***C***H_2_-CN), 47.1, 48.6 [N^α^-(CH_2_)_2_, N^β^-(CH_2_)_2_], 53.3 (βC), 61.7 (αC), 119.68, 119.72 (αCN, βCN), 172.9 (COOH); Anal. Calcd for C_15_H_20_O_2_N_6_: C, 56.95; H, 6.37; N, 26.56. Found: C, 56.99; H, 6.37; N, 26.67; α[D]^29.1^^°C^ = -71° (±1°, c=2 in acetone). Monocrystals of *rac-***3a** prepared in a separate procedure were subjected to X-ray analysis [31].

*N,N’,N_,_N’-tetrakis(cyanoethyl)-L-α,γ-diaminobutyric acid* (**3b**): Yield: 7.1 g (21.1%); semisolid; ESI MS 353 (M+23); ^1^H-NMR (DMSO) δ 1.61 (m, 2H, βCH_2_), 2.47 (t, *J*=6.95 Hz, 2H, γCH_2_), 2.58 [m, 8H, CH_2_CN), 2.75 [m, 8H, N-(CH_2_)_2_], 3.67 (t, *J* = 6.00 Hz, 1H, αCH), 11.55 (s, 1H, COOH); ^13^C-NMR δ 15.6, 18.0 (α***C***H_2_-CN, γ***C***H_2_-CN), 31.1 (βC), 47.8, 48.4 [N^α^-(CH_2_)_2_, N_γ_-(CH_2_)_2_], 51.6 (γC), 65.1 (αC), 119.1, 120.0 (αCN, γCN), 174.6 (COOH); Anal. Calcd for C_16_H_22_O_2_N_6_: C, 58.16; H, 6.71; N, 25.43. Found: C, 57.95; H, 6.62; N, 25.23; α[D]^29.1^^°^^C^ = -64° (±1°, c = 2 in acetone).

*N,N’,N,N’-tetrakis(cyanoethyl)-L-ornithine* (**3c**): Yield: 8.2 g (23.6%); semisolid; ESI MS 345 (M+1), 367 (M+23); ^1^H-NMR (CDCl_3_) δ 1.40, 1.54, 1.68 (3m, 4H, γCH_2_, βCH_2_), 2.50 (m, 2H, δCH_2_), 2.59 [m, 8H, CH_2_CN), 2.76, 2.88 [2m, 8H, N-(CH2)2], 3.34 (dd, *J* = 4.45 Hz, 1H, αCH); ^13^C-NMR δ 15.5, 17.4 (α***C***H_2_-CN, δ***C***H_2_-CN), 23.5 (γC), 27.1 (βC), 46.9, 48.4 [N^α^-(CH_2_)_2_, N^δ^-(CH_2_)_2_], 51.9 (δC), 62.5 (αC), 119.86, 119.91 (αCN, δCN), 174.2 (COOH); Anal. Calcd for C_17_H_24_O_2_N_6_: C, 59.28; H, 7.02; N, 24.40. Found: C, 59.04; H, 6.96; N, 24.19; α[D]^29.1^^°^^C^ = -65.2° (±1°, c = 2 in acetone).

*N,N’,N,N’-tetrakis(cyanoethyl)-L-lysine* (**3d**): Yield: 8.5 g (23.8%); semisolid; ESI MS 359 (M+1), 381 (M+23); ^1^H-NMR (CDCl_3_) δ 1.52, 1.68, 1.85 (3m, 6H, γCH_2_, δCH_2_, βCH_2_), 2.52 (m, 10H, CH_2_CN, εCH_2_), 2.85, 3.04 [2m, 8H, N-(CH2)2], 3.38 (dd, *J* = 5.34 Hz, 1H, αCH); ^13^C-NMR δ 16.9, 18.4 (α***C***H_2_-CN, ε***C***H_2_-CN), 24.1 (γC), 26.9 (δC), 29.6 (βC), 47.5, 49.5 [N^α^-(CH_2_)_2_, N^ε^-(CH_2_)_2_], 52.9 (εC), 63.4 (αC), 118.77, 118.84 (αCN, εCN), 177.2 (COOH); Anal. Calcd for C_18_H_26_O_2_N_6_: C, 60.3; H, 7.3; N, 23.4. Found: C, 60.08; H, 7.26; N, 23.15; α[D]^28.6^^°C^ = -63° (±1°, c = 2 in acetone).

#### 3.5.2. Synthesis of *N,N*-bis(cyanoethyl)-L-*α,β*-diamino propionic acid (**11**)

L-α,β-Diaminopropionic acid hydrochloride (14.05 g, 0.1 mol) was suspended in MeOH (100 mL), followed by slow addition of NaOH (8 g) in MeOH (100 mL). The reaction mixture was cooled to 0 °C in an ice-water bath. Then CH_2_=CH-CN (26.33 mL, 0.4 mol) was added in small portions and vigorous stirring was continued overnight, followed by addition of concentrated HCl (16.9 mL, 0.2 mol). The resulting white precipitate was collected on a filter and recrystallized from a MeOH-H_2_O mixture (1:1, v/v) to give 16.9 g (66.92%) of **11**. Yield: 16.9g (66.9%); mp 180-182 °C (dec.); ESI MS 211 (M+1), 233 (M+23), 265 (M+23+32); ^1^H-NMR (D_2_O) δ 3.03, 3.07 (2t, *J* = 6.74 Hz, 4H, α, βCH_2_CN), 3.45-3.65 (bm, 6H, N-CH_2_), 4.06 (dd, *J* = 5.50 Hz, 1H, αCH); ^13^C-NMR δ 15.3, 15.8 (α, β***C***H_2_-CN), 42.74, 43.78 (α, βNH-CH_2_-), 45.8 (βC), 56.4 (αC), 117.7, 118.0 (α, βCN), 170.2 (COOH); Anal. Calcd for C_9_H_15_O_2_N_4_Cl: C, 43.82; H, 6.12; N, 22.71; Cl, 14.37. Found: C, 43.66; H, 6.20; N, 22.55; Cl, 14.41; α[D]^24.5^^°C^ = +24.2 ± 1° (c = 2 in 1 M HCl).

#### 3.5.3. General procedure for the catalytic reduction of the nitriles **2a-d** to the tris-*N*-propylamino derivatives **4a**-**d** and the synthesis of the *Z*-protected derivatives **4e-h**

To a methanolic solution (150 mL) containing a tris-*N*-cyanoethylated derivative of basic amino acid **2a**-**d** (5 g, ca. 15 mmol) and NaOH (0.64 g, 16 mmol), Raney-Ni suspension (5 g) was added. The resulting mixture was agitated at room temperature for 14-16 h under 90 psi (6 bar) of H_2_ pressure. The progress of the reaction was monitored by TLC using 1% ninhydrin in ethanol. After completion of the reaction, the catalyst was separated by filtration on Celite and the remaining solution was evaporated to dryness giving the compounds **4a**-**d** as white hygroscopic oils in almost quantitative yields. The crude products were used for the next step without further purification.

*N,N,N’-tris(3-aminopropyl)-L-α,β-diaminopropionic acid* (**4a**): Yield: ~100%; ESI MS 298 (M+23), 330 (M+23+32); ^1^H-NMR (D_2_O) δ 1.50-1.70 (m, 6H, C-CH_2_-C), 2.50-2.70 (bm, 14H, N-CH_2_), 3.15 (dd, *J* = 5.65 Hz, 1H, αCH), 3.34 (s, 6H, NH_2_); ^13^C-NMR δ 28.3 (β***C***H_2_-CH_2_NH_2_), 28.9 (α***C***H_2_-CH_2_NH_2_), 38.9 (αCH_2_NH_2_), 39.3 (βCH_2_NH_2_), 44.9 (NH^α^-CH_2_), 49.1 [N^β^-(CH_2_)_2_], 62.0 (αC), 181.8 (COO^-^).

*N,N_,_N’-tris(3-aminopropyl)-L-α,γ-diaminobutyric acid* (**4b**): Yield: ~100%; ESI MS 344 (M+23+32); ^1^H-NMR (D_2_O) δ 1.55-1.75 (bm, 8H, C-CH_2_-C), 2.40-2.80 (bm, 14H, N-CH_2_), 2.98 (dd, *J* = 5.33 Hz, 1H, αCH), 3.35 (s, 6H, NH_2_); ^13^C-NMR δ 28.8 (γ***C***H_2_-CH_2_NH_2_), 29.0 (α***C***H_2_-CH_2_NH_2_), 32.0 (βC), 38.9 (αCH_2_NH_2_), 39.4 (γCH_2_NH_2_), 45.1 (NH^α^-CH_2_), 49.1 [N^γ^-(CH_2_)_2_], 50.9 (γC), 62.67 (αC), 182.3 (COO^-^).

*N,N,N’-tris(3-aminopropyl)-L-ornithine* (**4c**): Yield: ~100%; ESI MS 326 (M+23); ^1^H-NMR (D_2_O) δ 1.39 (m, 4H, β,γCH_2_), 1.53 (m, 6H, C-CH_2_-C), 2.30-2.60 (bm, 14H, N-CH_2_), 2.95 (dd, *J* = 6.02 Hz, 1H, αCH), 3.28 (s, 6H, NH_2_); ^13^C-NMR δ 21.5 (α***C***H_2_-CH_2_NH_2_), 28.1 (δ***C***H_2_-CH_2_NH_2_), 30.7 (γC), 31.6 (βC), 38.4 (αCH_2_NH_2_), 38.9 (δCH_2_NH_2_), 44.6 (NH^α^-CH_2_), 48.6 [N^δ^-(CH_2_)_2_], 50.4 (δC), 63.4 (αC), 182.3 (COO^-^).

*N,N,N’-tris(3-aminopropyl)-L-lysine* (**4d**): Yield: ~100%; ESI MS 318 (M+1); ^1^H-NMR (D_2_O) δ 1.50 (m, 2H, γCH_2_), 1.81 (m, 2H, δCH_2_), 2.06 (m, 2H, βCH_2_), 2.15 (m, 6H, C-CH_2_-C), 3.10 (t, *J* = 7.7 Hz, 6H, C***H***_2_NH_2_), 3.20-3.30 (bm, 8H, N^ε^-CH_2_, NH^α^-CH_2_), 3.34 (s, 6H, NH_2_), 3.97 (dd, *J* = 5.09 Hz, 1H, αCH); ^13^C-NMR δ 21.5 (α***C***H_2_-CH_2_NH_2_), 21.7 (ε***C***H_2_-CH_2_NH_2_), 22.9(γC), 23.9 (δC), 28.6 (βC), 36.6 (εCH_2_NH_2_), 36.7 (αCH_2_NH_2_), 43.9 (NH^α^-CH_2_), 50.1 [N^ε^-(CH_2_)_2_], 52.7 (εC), 60.7 (αC), 181.5 (COO^-^).

*N,N-bis(3-aminopropyl)-L-α,β-diaminopropionic acid* (**12**): Yield: ~100%; ESI MS 272 (M+NH_4_^+^+ 2H_2_O), 273 (M+Na^+^+MeOH), 275 (M+K^+^+H_2_O); ^1^H-NMR (D_2_O) δ 1.60 (m, 4H, C-CH_2_-C), 2.50-2.75 (bm, 10H, βCH_2_, N-CH_2_), 3.19 (t, *J* = 6.46 Hz, 1H, αCH), 3.35 (s, 6H, NH); ^13^C=NMR δ 31.9, 32.1 (α, β***C***H_2_-CH_2_NH_2_), 38.9 (α, β***C***H_2_NH_2_), 45.4, 46.4 (NH-CH_2_), 51.4 (βCH_2_), 63.2 (αC), 181.44 (COO^-^).

#### 3.5.4. General synthesis of the *Z*-protected derivatives **4e**-**h**

To the crude products from the previous reaction (8.5-9.5 g, ca. 15 mmol, 100%), a dioxane-water mixture (1:1, v/v, 100 mL) was added, followed by addition of benzyloxycarbonyl chloride (12 mL, 85.7 mmol). The pH value of the mixture was maintained between 9 and 10 by adding 1M NaOH (88 mL). The reaction mixture was cooled in an ice-water bath; after addition of substrates, it was allowed to warm to room temperature and was stirred overnight. Then the aqueous phase was washed three times with diethyl ether (50 mL; the ether extracts were discarded), acidified using 10% citric acid, and extracted three times with chloroform (100 mL). The CHCl_3_ layers were combined, dried over Na_2_SO_4_ overnight and concentrated *in vacuo*. The residue was purified by flash chromatography (CHCl_3_:MeOH 8:1) to give **4e-h** (58.7-69%) as pale colored gums.

*N^α^-benzyloxycarbonyl-N_,_N_,_N’-tris(benzyloxycarbonyl-3-aminopropyl)-*L-*α,β-diaminopropionic acid* (**4e**): Yield: 9.05g (58.7%); yellow gum; ESI MS 826 (esterification in MeOH in the spectrometer!); ^1^H-NMR (DMSO, 350K) δ 1.60-1.80 (m, 6H, C-CH_2_-C), 2.80-3.30 (bm, 12H, N-CH_2_), 3.60 (m, 2H, βCH_2_), 4.30 (m, 1H, αCH), 5.04 (s, 8H, CH_2_Ar), 7.29 (m, 20H, Ar-H); ^13^C-NMR δ 28.5, 29.5 (C-***C***H_2_-C), 39.79, 39.81 (CH_2_NH), 43.7 (N^α^-CH_2_), 49.6 [N^β^-(CH_2_)_2_], 51.8 (βC), 59.1 (αC), 65.4, 66.8 (CH_2_-Ar), 127.7, 127.8, 128.3, 137.0 (C_Ar_), 155.1, 156.4 (O-CO-N-), 172.4 (COOH); α[D]^26.6^^°C^ = -10.30 ± 0.5° (c=2 in acetone).

*N^α^-benzyloxycarbonyl-N,N_,_N’-tris(benzyloxycarbonyl-3-aminopropyl)-L-α,γ-diaminobutyric acid* (**4f**): Yield: 10.25 g (69%); colorless gum; ESI MS 839 (M’ + 1, esterification in MeOH in the spectrometer!), 916 (M + C_7_H_7_^+^); ^1^H-NMR (DMSO, 350K) δ 1.49-1.66 (bm, 8H, C-CH_2_-C), 3.0-3.6 (bm, 15H, N-CH_2_) 4.15 (m, 1H, αCH), 5.02 (s, 8H, CH_2_-Ar), 7.33 (bs, 20H, Ar-H); ^13^C-NMR δ 25.6, 26.1 (C-***C***H_2_-C), 28.5 (βC), 38.5 (CH_2_NH), 45.0 (NH^α^-CH_2_), 50.7 [N^γ^-(CH_2_)_2_], 51.0 (γC), 60.1 (αC) 64.8, 66.0 (CH_2_-Ar), 126.8, 127.0, 127.8, 136.9 (C_Ar_), 155.6 (O-CO-N-), 172.3 (COOH); α[D]^26.3^^°C^ = -10.1 ± 0.5° (c = 2 in acetone).

*N^α^-benzyloxycarbonyl-N,N_,_N’-tris(benzyloxycarbonyl-3-aminopropyl)-L-ornithine* (**4g**):Yield: 8.16 g (56.6%); pale yellow gum; ESI MS 840 (M+1), 854 (M’ + 1, esterification in MeOH in the spectrometer!); ^1^H-NMR (DMSO, 350K) δ 1.48, 2.31 (2m, 10H, C-CH_2_-C, β, γCH_2_), 2.90-3.80 (bm, 14H, N-CH_2_), 4.24 (m, 1H, αCH), 5.02, 5.18 (s, 8H, CH_2_Ar), 7.31 (m, 20H, Ar-H); ^13^C-NMR δ 23.2 (γC), 26.8 (C-***C***H_2_-C), 29.4 (βC), 38.7 (CH_2_NH), 45.1 (N^α^-CH_2_), 50.8 [N^δ^-(CH_2_)_2_], 52.8 (δC), 59.8 (αC), 65.1, 66.3 (CH_2_-Ar), 127.8, 128.3, 137.3 (C_Ar_), 155.4, 156.0 (O-CO-N-), 171.5 (COOH); α[D]^26.5^^°C^ = -10.12 ± 0.5° (c = 2 in acetone).

*N^α^-benzyloxycarbonyl-N,N,N’-tris(benzyloxycarbonyl-3-aminopropyl)-L-lysine* (**4h**): Yield: 8.50 g (62.27%); yellow gum; ESI MS 854 (M+1), 944 (M + C_7_H_7_^+^), 1034 (M-1+2C_7_H_7_^+^); ^1^H-NMR (DMSO, 350K) δ 1.15-2.0 (bm, 12H, β, γ, δCH_2_, C-CH_2_-C), 2.50, 3.10 (2 bm, 14H, NCH_2_), 4.10 (m, 1H, αCH), 4.97, 5.03 (bs, 8H, CH_2_Ar), 7.29 (bm, 20H, Ar-H); ^13^C-NMR δ 22.6 (γC), 28.6 (δC), 28.8 (C-***C***H_2_-C), 29.8 (βC), 37.6, 38.5 (CH_2_NH), 44.5 (N^α^-CH_2_), 50.6 [N^ε^-(CH_2_)_2_], 52.2 (εC), 58.4 (αC), 66.3, 66.9 (CH_2_-Ar), 127.9, 128.3, 136.7, 136.8 (C_Ar_), 156.6, 157.1 (O-CO-N-), 172.7 (COOH); α[D]^23.6^^°C^ = -10.33 ± 0.5° (c = 2 in acetone).

*N,N^β^-bis(benzyloxycarbonyl)-N^α^,N^β^-bis(benzyloxycarbonyl-3-aminopropyl)-L-α,β-diaminopropionic* acid (**13**): Yield: 11.1g (61.83%); amber-colored gum; ESI MS 777 (M+23, CHCl_3_); ^1^H-NMR (DMSO, 350K) δ 1.60-1.70 (m, 4H, C-CH_2_-C), 2.96 (m, 4H, C***H***_2_NH), 3.20, 3.30 (2 m, 4H, N-CH_2_), 3.70 (m, 2H, βCH_2_), 4.25 (m, 1H, αCH), 5.04 (s, 8H, CH_2_Ar), 7.29 (m, 20H, Ar-H); ^13^C-NMR δ 28.7 (C-***C***H_2_-C), 38.8 (CH_2_NH), 45.8 (NH-CH_2_), 47.1 (βC), 59.2 (αC), 64.9, 66.0 (CH_2_-Ar), 128.1, 128.7, 137.79, 137.82 (C_Ar_), 155.7, 156.5 (O-CO-N-), 171.39 (COOH); α[D]^24.5^^°C^ = -52.50 ± 1° (c = 2 in acetone).

#### 3.5.5. General procedure for the reduction of the nitriles to the corresponding tetrakis-N-propylamino derivatives **5a**-**d**

To a derivative **3a**-**d** (5 g, ca. 15 mmol) dissolved in dry THF (250 mL), 10 M solution of BH_3_ × SMe_2_ (Aldrich, 15.8 mL) were added. The reaction mixture was heated to reflux and stirred vigorously overnight. After cooling to room temperature, 1M HCl in MeOH (94.8 mL) and additional MeOH (100 mL) were added. The mixture was evaporated *in vacuo*, the oily residue was treated three times with 50 mL of MeOH and evaporated to dryness, to give 8.5-9 g (ca. 100%) of a white, very hygroscopic foam. The elemental analysis shows a mixture of penta- and hexahydrochlorides of **5a-d**. 

*N,N’,N,N’-tetrakis(3-aminopropyl)-L-α,β-diaminopropanol* (**5a**): Yield: 8.9g (~100%); ESI MS 319 (M+1); ^1^H-NMR (D_2_O) δ 1.90-2.10 (m, 8H, C-CH_2_-C), 3.00-3.70 (3m, 20H, N-CH_2_, C***H***_2_OH), 3.90 (2m, 1H, αCH); ^13^C-NMR δ 26.7, 29.3 (αC-***C***H_2_-C, βC-***C***H_2_-C), 37.6, 38.0 (CH_2_NH_2_), 48.7, 57.8 [N^α^-(CH_2_)_2_, N_β_-(CH_2_)_2_], 58.34 (βC), 59.2 (αC), 61.7 (CH_2_OH); α[D]^26.5^ ^°C^ = +15.6° (±1°, c = 2 in 1M HCl).

*N,N’,N,N’-tetrakis(3-aminopropyl)-L-α,γ-diaminobutanol* (**5b**): Yield: 8.8g (~100%); ESI MS 353 (M+23); ^1^H-NMR (D_2_O) δ 1.60-2.2 (bm, 10H, βCH_2_, C-CH_2_C), 2.80-3.60 (bm, 20H, N-CH_2_, C***H***_2_OH), 3.87 (m, 1H, αCH); ^13^C-NMR δ 21.7, 22.9 (C-***C***H_2_-C), 28.8 (βC), 36.5, 36.6 (CH_2_NH_2_), 49.0, 50.1 [N^α^-(CH_2_)_2_, N^γ^-(CH_2_)_2_], 52.1 (γC), 57.35 (αC), 61.9 (CH_2_OH); α[D]^29.1^^°C^ = +12.7° (±1°, c=2 in 1M HCl).

*N,N’,N,N’-tetrakis(3-aminopropyl)-L-α,δ-diaminopentanol* (**5c**): Yield: 8.7g (~100%); ESI MS 347 (M+1); ^1^H-NMR (CDCl_3_) δ 1.55, 1.80, 2.10 (3m, 12H, β, γCH_2_, C-CH_2_-C), 2.90-3.40 (bm, 20H, N-CH_2_, C***H***_2_OH), 3.95 (2m, 1H, αCH); ^13^C-NMR δ 21.8 (C-***C***H_2_-C), 22.9 (γC), 27.9 (βC), 36.6, 36.7 (CH_2_NH_2_), 50.0 [α, δN-(CH_2_)_2_], 52.9 (δC), 57.8 (αC), 61.6 (CH_2_OH); α[D]^29.1^^°C^ = +12.3° (±1°, c=2 in 1M HCl).

*N,N’,N,N’-tetrakis(3-aminopropyl)-L-α,ε-diaminohexanol* (**5d**): Yield: 8.9 g (~100%); ESI MS 361 (M+1), 383 (M+23); ^1^H-NMR (CDCl_3_) δ 1.50-2.30 (3m, 14H, β, γ, δCH_2_, C-CH_2_-C), 2.90-3.40 (bm, 20H, N-CH_2_, C***H***_2_OH), 3.95 (m, 1H, αCH); ^13^C-NMR δ 22.0 (C-***C***H_2_-C), 23.3 (γC), 23.9 (δC), 26.3 (βC), 36.9, 37.3 (CH_2_NH_2_), 49.2, 50.21 [α, ε N-(CH_2_)_2_], 53.2 (εC), 58.9 (αC), 61.8 (CH_2_OH); α[D]^28.6^^°C^ = +13.2 ± 1°, (c = 2 in 1M HCl).

#### 3.5.6. Preparation of dendrimeric compounds **7e-7h**

To benzylamine (3.21 g, 3.3 mL = 30 mmol) dissolved in dry THF (15 mL), **4g** (2.55 g, 3 mmol), HOBt (0.46 g, 3 mmol) and DCC (0.63 g, 3 mmol) were added, the mixture was stirred for 24 h and the solvent was evaporated *in vacuo*. The residue was dissolved in CHCl_3_ (50 mL) and washed consecutively with 10% Na_2_CO_3_, H_2_O, 1% citric acid and saturated NaCl solution, then dried over Na_2_SO_4_ and evaporated to dryness: yield 2.07 g (74.3%) of the amide **6g** as a light yellow gum.

*N,N_,_N’-tris(benzyloxycarbonyl-3-aminopropyl)-L-diaminoalanine benzylamide* (**6e**): C_51_H_60_O_9_N_6_, Yield: 1,92g (65.3%); yellow gum; ESI MS 901 (M+H^+^), 923 (M+Na^+^); ^1^H-NMR (DMSO, 350K) δ 1.66-1.85 (m, 6H, C-CH_2_-C), 2.79-3.38 (bm, 12H, N-CH_2_), 3.63 (m, 2H, βCH_2_), 4.23 (s, 2H, Ar-C***H***_2_-NH), 4.3 (m, 1H, αCH), 5.05, 5.09 (2s, 8H, CH_2_-Ar), 7.2-7.3 (bm, 25H, Ar-H); ^13^C-NMR δ 28.6, 29.1 (C-***C***H_2_-C), 40.1 (CH_2_NH), 42.3 (Ar-***C***H_2_-NH), 43.7 (N_α_-**C**H_2_), 49.5 [N_β_-(CH_2_)_2_], 51.1 (βC), 59.6 (αC), 65.1, 66.3 (CH_2_-Ar), 127.7, 127.8, 128.2 (CH_Ar_), 136.6, 136.9 (C_Ar_), 155.0, 156.2 (O-CO-N-), 173.1 (CONH).

*N,N_,_N’-tris(benzyloxycarbonyl-3-aminopropyl)-L-diaminobutyric benzylamide* (**6f**): Yield 2.15 g (70.1%); pale yellow gum; ESI MS 915 (M+H^+^), 937 (M+Na^+^); ^1^H-NMR (DMSO, 350K) δ 1.45-1.7 (3m, 8H, βCH_2_, C-CH_2_-C), 2.9-3.5 (bm, 14H, N-C***H***_2_), 4.2 (s, 2H, Ar-C***H***_2_-NH), 4.28 (m, 1H, αCH), 5.07, 5.11 (2s, 8H, CH_2_-Ar), 7.2-7.3 (bm, 25H, Ar-H). ^13^C-NMR δ 25.5, 26.4 (C-***C***H_2_-C), 29.1 (βC), 38.5 (CH_2_NH), 42.4 (Ar-***C***H_2_-NH), 44.5 (NH_α_-CH_2_), 50.3 [N_γ_-(CH_2_)_2_], 51.1 (γC), 59.8 (αC), 65.0, 66.1 (CH_2_-Ar), 126.9, 127.0, 127.8, 128.0 (CH_Ar_), 136.8, 137.0 (C_Ar_), 155.3, 156.2 (O-CO-N-), 173.0 (CONH).

*N,N_,_N’-tris(benzyloxycarbonyl-3-aminopropyl)-L-ornithine benzylamide* (**6g**): Yield: 74.3%; C_53_H_64_O_9_N_6_, pale yellow gum; ESI MS 929 (M+1), 951 (M+23), 967 (M+39), 1,019 (M + 91 = C_7_H_7_^+^); ^1^H-NMR (DMSO, 350K) δ 1.40 - 2.30 (2m, 10H, C-CH_2_-C, β, γCH_2_,), 2.85-3.70 (bm, 14H, N-CH_2_), 4.22 (s, 2H, Ar-C***H***_2_-NH), 4.31 (m, 1H, αCH), 5.05, 5.20 (s, 8H, CH_2_Ar), 7.29 (m, 25H, Ar-H); ^13^C- NMR δ 23.2 (γC), 26.3 (C-***C***H_2_-C), 29.6 (βC), 38.6 (CH_2_NH), 42.2 (Ar-***C***H_2_-NH), 44.2 (N_α_-CH_2_), 50.6 [N_δ_-(CH_2_)_2_], 52.6 (δC), 59.7 (αC), 65.0, 66.3 (O***C***H_2_-Ar), 127.6, 127.7, 128.3, 137.3 (C_Ar_), 155.2, 156.1 (O-CO-N-), 171.3 (CONH).

*N,N_,_N’-tris(benzyloxycarbonyl-3-aminopropyl)-L-lysine benzylamide* (**6h**): Yield: 78.6%; C_54_H_66_O_9_N_6_, yellow gum; ESI MS 943 (M+1), 965 (M+23), 981 (M+39) 1,033 (M+91 = C_7_H_7_^+^); ^1^H-NMR (DMSO, 350K) δ 1.2-2.1 (bm, 12H, β, γ, δCH_2_, C-CH_2_-C), 2.52, 3.12 (2 bm, 14H, NCH_2_), 4.19 (m, 1H, αCH), 4.26 (s, 2H, Ar-C***H***_2_-NH), 5.03, 5.1 (bs, 8H, CH_2_Ar), 7.15-7.3 (bm, 25H, Ar-H); ^13^C-NMR δ 22.7(γC), 28.4 (δC), 28.5 (C-***C***H_2_-C), 29.4 (βC), 37.4, 38.1 (CH_2_NH), 42.1 (Ar-***C***H_2_-NH), 43.9 (N_α_-CH_2_), 49.9 [N_ε_-(CH_2_)_2_], 52.1 (εC), 58.5 (αC), 66.4, 66.8 (O***C***H_2_-Ar), 127.9, 128.4, 136.8, 136.9 (C_Ar_), 156.6, 156.9 (O-CO-N-), 171.9 (CONH).

A) **6g** (1.5 g, 1.6 mmol) dissolved in MeOH (25 mL) was stirred for 24 h with 10% Pd/C (150 mg) under an atmospheric pressure of H_2_. Then the catalyst was separated on Celite® and washed with MeOH. The collected methanol fractions were evaporated to dryness yielding 0.59 g (93.1%) of deprotected **6g** as a white waxy solid. To the solution of deprotected **6g** in DMF (25 mL), (2-Cl-Z)Lys(Boc) (2.04 g, 4.95 mmol), HOSu (0.57 g, 4.95 mmol) and DCC (1.02 g, 4.95 mmol) were added. The mixture was stirred for 24 h (until disappearance of free amino groups in the ninhydrin test), filtered and evaporated *in vacuo*. The residue was dissolved in CHCl_3_ (50 mL) and washed with 10% Na_2_CO_3_, H_2_O, 1% citric acid, dried over Na_2_SO_4_ overnight and evaporated to dryness. The residue was purified by flash chromatography (CHCl_3_-MeOH 8:1) to give 1.7 g (71.3%) of Boc-protected **7g** as a dark yellow resin.
B) Boc-protected **7g** (0.5 g, 0.32mmol) was dissolved in CH_2_Cl_2_ (5 mL) and trifluoroacetic acid (TFA, 5 mL) was added. The reaction mixture was stirred at room temperature for 3 h. Then the reaction mixture was evaporated *in vacuo*, the residue was dissolved in ethyl acetate (5 mL) and evaporated (3 times) and then in diethyl ether (5 mL) and evaporated (twice) – to remove all remaining trifluoroacetic acid. Trifluoroacetate ions were replaced by chlorides by dissolving the oily residue in HCl-saturated ethyl acetate and evaporation *in vacuo* (four times) to give 470 mg (99%) of **7g** hexahydrochloride as a yellow glassy gum.

*N,N,N’-tris[(N^α^-2-chlorobenzyloxycarbonyl)-L-lysil-3-aminopropyl]-L-diaminoalanine benzylamide pentahydrochloride* (**7e**): C_61_H_87_O_10_N_12_Cl_3_·5HCl; Yield 0.99g (97.5%); ESI MS 627 (M+2H^+^)^2+^ - base peak, 647 (M+H^+^+Na^+^+H_2_O)^2+^, 1,253 (M+H^+^); ^1^H-NMR (DMSO, 350K) δ1.1 – 2.1 (4bm, 24H, C-C***H***_2_-C, β, γ,δCH_2_ G-1 *Lys*), 2.6-3.5 (3 bm, 20H, αNH-C***H***_2_, βN-(CH_2_)_2_, C***H***_2_-NH core, εCH_2_ G-1 *Lys*), 3.53 (m, 1H, αCH core), 4.18 (m, 3H, αCH G-1 *Lys*), 4.30 (Ar-C***H***_2_-NH), 5.11, 5.18 (2bs, 6H, Ar-CH_2_O- from *2-Cl-Z*), 7.2-7.4 (bm; 17H, Ar-H); ^13^C-NMR δ 22.2 (γC G-1 *Lys*), 25.1 (C-***C***H_2_-C core), 29.7 (δC G-1 *Lys*), 31.0 (βC G-1 *Lys*), 38.5 (εC G-1 *Lys*), 40.1 (***C***H_2_NH core), 43.4 (Ar***C***H_2_-NH), 44.6 (N_α_-***C***H_2_ core), 50.9 [N_β_-(***C***H_2_)_2_ core], 52.7 (βC core), 55.4 (αC G-1 *Lys*), 59.5 (αC core *Lys*), 62.1, 62.3 (Ar-***C***H_2_-O), 126.5, 126.6, 127.4, 127.8, 128.7, 129.0, 129.1, 129.2 (CH_Ar_), 132.0 (C_Ar_-Cl), 133.7 (***C***_Ar_-CH_2_-NH), 134.1 (***C***_Ar_-CH_2_-O), 155.6, 156.7 (O-CO-NH-), 171.3 (CONH G-1 *Lys*), 172.2 (CONH core); Anal. Calcd for C_61_H_87_O_10_N_12_Cl_3_·5HCl, (12 days/P_2_O_5_): C, 51.0; H, 6.45; N, 11.70; Cl, 19.73. Found: C, 50.67; H, 6.51; N, 11.43; Cl, 19.5.

*N,N,N’-tris[(N^α^-2-chlorobenzyloxycarbonyl)-L-lysil-3-aminopropyl]-L-diaminobutyro benzylamide pentahydrochloride* (**7f**): C_62_H_89_O_10_N_12_Cl_3_·5HCl; Yield 1.0g (98.3%); hygroscopic yellow gum; ESI MS 634 (M+2H^+^)^2+^- base peak, 654 (M+H^+^+Na^+^+H_2_O)^2+^, 1,267 (M+H^+^); ^1^H-NMR (DMSO, 350K) δ 1.1 – 2.1 (bm, 26H, C-C***H***_2_-C, βCH_2_ core and β, γ,δCH_2_ G-1 *Lys*), 2.5-3.55 (2 bm, 20H, αNH-C***H***_2_, γ N-(CH_2_)_2_, C***H***_2_-NH core, εCH_2_ G-1 *Lys*), 3.68 (m, 1H, αCH core), 4.15 (m, 3H, αCH G-1 *Lys*), 4.28 (Ar-C***H***_2_-NH), 5.09, 5.15 (2bs, 6H, Ar-CH_2_O- grup *2-Cl-Z*), 7.2-7.4 (bm; 17H, Ar-H); ^13^C-NMR δ 22.1 (γC G-1 *Lys*), 25.3 (C-***C***H_2_-C core), 29.1 (βC core), 29.6 (δC G-1 *Lys*), 31.1 (βC core *Lys*), 38.2 (εC G-1 *Lys*), 39.9 (***C***H_2_NH core), 43.2 (Ar***C***H_2_-NH), 44.9 (N_α_-***C***H_2_ core), 50.8 [N_γ_-(***C***H_2_)_2_ core], 53.1 (γC core), 55.3 (αC G-1 *Lys*), 59.7 (αC core), 62.2, 62.4 (Ar-***C***H_2_-O), 126.5, 126.7, 127.3, 127.8, 128.7, 129.0, 129.1, 129.2 (CH_Ar_), 131.9 (C_Ar_-Cl), 133.8 (***C***_Ar_-CH_2_-NH), 134.0 (***C***_Ar_-CH_2_-O), 155.7, 156.8 (O-CO-NH-), 171.4 (CONH G-1 *Lys*), 172.2 (CONH core); Anal. Calcd for C_62_H_89_O_10_N_12_Cl_3_·5HCl, (12 days/P_2_O_5_): C, 51.3; H, 6.52; N, 11.58; Cl, 19.54. Found: C, 50.96; H, 6.61; N, 11.31; Cl, 19.42.

*N,N,N’-tris[(N^α^-2-chlorobenzyloxycarbonyl)-L-lysil-3-aminopropyl]-L-ornithine benzylamide penta- hydrochloride* (**7g**): Yield: 99%; C_63_H_91_O_10_N_12_Cl_3_·5HCl, hygroscopic, glass-like gum; ESI MS 641 (M+2)^2+^, 1,281 (M+1), 1,299 (M+18+1); ^1^H-NMR (DMSO, 350K) δ 1.3 – 2.2 (bm, 28H, core β, γCH_2_, C-C***H***_2_-C, G1 β, γ,δCH_2_), 2.4-3.3 (bm, 20H, core αNH-C***H***_2_, δN-(CH_2_)_2_, C***H***_2_-NH, G1 εCH_2_), 3.60 (m, 1H, core αCH), 4.19 (m, 3H, G1 αCH), 4.38 (Ar-C***H***_2_-NH), 5.17 (m, 6H, G1 Ar-CH_2_O-), 7.2-7.4 (bm; 17H, Ar-H); ^13^C-NMR δ 22.4 (G1 γC), 23.1 (core γC), 27.6 (core C-***C***H_2_-C), 29.3 (G1 δC), 29.5 (core βC), 32.3(G1 βC), 36.3 (core ***C***H_2_NH), 39.8, 40.0 (G1 εC), 43.2 (Ar***C***H_2_-NH), 44.1 (core N_α_-***C***H_2_), 49.7 [core N_δ_-(***C***H_2_)_2_], 51.6 (core δC), 55.0, 55.2 (G1 αC), 58.8 (core αC), 66.5, 66.8 (Ar-***C***H_2_-O), 126.67, 126.7, 126.8, 127.6, 128.5, 128.9, 129.0, 129.3 (CH_Ar_), 132.8 (G1 C_Ar_-Cl), 133.9 (***C***_Ar_-CH_2_-NH), 134.4 (G1 ***C***_Ar_-CH_2_-O), 155.8, 156.0 (G1 O-CO-N-), 174.0 (core CONH), 175.2 (G1 CONH); Anal. Calcd for C_63_H_91_O_10_N_12_Cl_3_·5HCl: C, 51.65; H, 6.26; N, 11.47; Cl, 19.35. Found: C, 51.43; H, 6.45; N, 10.31; Cl, 19.05.

*N,N,N’-tris[(N^α^-2-chlorobenzyloxycarbonyl)-L-lysil-3-aminopropyl]-L-lysine benzylamide penta- hydrochloride* (**7h**): Yield: 99%; C_64_H_93_O_10_N_12_Cl_3_·5HCl, hygroscopic, glassy solid; ESI MS 648 (M+2)^2+^, 659 (M+1+23)^2+^ 1,295 (M+1), 1,313 (M+18+1); ^1^H-NMR (DMSO, 350K) δ 1.1–2.1 (bm, 30H, C-C***H***_2_-C, core and G1 β, γ,δCH_2_), 2.6-3.5 (2 bm, 20H, core αNH-C***H***_2_, εN-(CH_2_)_2_, C***H***_2_-NH, G1 εCH_2_), 3.65 (m, 1H, core αCH), 4.05 (m, 3H, G1 αCH), 4.33 (Ar-C***H***_2_-NH), 5.12 (m, 6H, G1 Ar-CH_2_O-), 7.2-7.4 (bm; 17H, Ar-H); ^13^C-NMR δ 21.9 (G1 γC), 22.1 (core γC), 24.5 (core δC) 25.6 (core C-***C***H_2_-C), 28.8 (core βC), 29.8 (G1 δC), 31.0 (G1 βC), 38.2 (G1 εC), 39.7 (core ***C***H_2_NH), 43.1 (Ar***C***H_2_-NH), 45.3 (core N_α_-***C***H_2_), 51.4 [core N_ε_-(***C***H_2_)_2_], 53.6 (core εC), 55.6 (G1 αC), 59.9 (core αC), 62.4, 62.5 (Ar-***C***H_2_-O), 126.5, 126.7, 127.1, 127.8, 128.7, 129.0, 129.1, 129.2 (CH_Ar_), 131.8 (G1 C_Ar_-Cl), 133.9 (***C***_Ar_-CH_2_-NH), 134.0 (G1 ***C***_Ar_-CH_2_-O), 156.7, 157.0 (G1 O-CO-N-), 171.5 (G1 CONH), 172.0 (core CONH); Anal. Calcd for C_64_H_93_O_10_N_12_Cl_3_*5HCl: C, 51.9; H, 6.67; N, 11.36; Cl, 19.17. Found: C, 51.63; H, 6.8; N, 11.19; Cl, 18.93.

#### 3.5.7. Preparation of the dendrimeric compounds **9a-d**

**5c** (450 mg, 0.72 mmol) was suspended in DMF (15 mL) with addition of N(Et)_3_ (2.40 mL, 17.28 mmol) and stirred at 40 °C until all of **5c** was dissolved. Then (Boc)-L-Phe-2,4,5-trichlorophenyl ester 1.76 g (3.96 mmol, 5.5 eq) was added and the reaction mixture was stirred at room temperature for 7 days until the complete disappearance of free amino groups in the ninhydrin test. The solution was evaporated *in vacuo*, the residue was dissolved in CHCl_3_ (50 mL) and washed with 20% K_2_CO_3_ and saturated NaCl solution, dried over Na_2_SO_4_ overnight, filtered and evaporated to dryness. The residue was purified by flash chromatography (CHCl_3_-MeOH, 8:1) to give 520 mg of **9c** (54%) as a white foam.

*N,N',N,N’-tetrakis(Boc-L-phenylalanyl-3-aminopropyl)-L-α,δ-diaminopropanol* (**9a**): Yield 2.76g (66%); C_71_H_106_O_13_N_10_; white foam; ESI MS 654 (M+2H^+^)^2+^, 1,307 (M+H^+^); ^1^H-NMR (DMSO, 350K) δ1.2-1.3 (m, 36H, Boc C-CH_3_), 1.47 (m, 8H, C-CH_2_-C), 2.33 (m, 4H, N_β_-CH_2_), 2.39 – 2.43 (m, 6H, βCH_2_, N_α_-CH_2_), 2.83, 2.93 (dd, *J* 5.3, 8.5 Hz, 8H, Ar-CH_2_), 3.18 (m, 8H, C***H***_2_-NH), 3.42 (m, 2H, C***H***_2_OH), 3.86 (m, 1H, αCH core), 4.23 (m, 4H, αCH *Phe*), 7.2 (m; 20H, Ar-H). ^13^C-NMR δ 21.2 (C-***C***H_2_-C), 28.2 (Boc C-***C***H_3_), 36.8, 36.9 (α, βCH_2_NH core), 50.0 [α, βN-(***C***H_2_)_2_], 52.8 (βC), 58.4 (αC), 62.0 (CH_2_OH), 77.7 (Boc ***C***-CH_3_), 125.2, 125.6, 127.6, 127.8, 128.7, 128.8 (CH_Ar_), 137.6, 138.5 (C_Ar_), 154.4, 154.8 (O-CO-NH), 172.2 (CONH *Phe*).

*N,N',N,N’-tetrakis(Boc-L-phenylalanyl-3-aminopropyl)-L-α,δ-diaminobutanol* (**9b**): Yield 3.9 g (71%); C_72_H_108_O_13_N_10_; ESI MS 662 (M+2H^+^)^2+^, 1,321 (M+H^+^); ^1^H-NMR (DMSO, 350K) δ1.2-1.3 (m, 36H, Boc C-CH_3_), 1.46 (m, 10H, βCH_2_, C-CH_2_-C), 2.35 (m, 6H, N_γ_-CH_2_), 2.38 (m, 4H, N_α_-CH_2_), 2.82, 2.94 (dd, *J* 5.3, 8.5 Hz, 8H, Ar-CH_2_), 3.15 (m, 8H, C***H***_2_-NH), 3.39 (m, 2H, C***H***_2_OH), 3.96 (m, 1H, αCH core), 4.22 (m, 4H, αCH *Phe*), 7.2 (m; 20H, Ar-H); ^13^C-NMR δ 21.6 (C-***C***H_2_-C), 27.8 – 28.4 (βC, Boc C-***C***H_3_), 36.6, 36.7 (α, γCH_2_NH core), 50.1 [α, γN-(***C***H_2_)_2_], 52.6 (γC), 57.7 (αC), 61.7 (CH_2_OH), 77.8 (Boc ***C***-CH_3_), 125.2, 125.7, 127.6, 127.7, 128.6, 128.8 (CH_Ar_), 137.7, 138.6 (C_Ar_), 154.3, 154.5 (O-CO-NH), 171.3 (CONH *Phe*).

*N,N',N,N’-tetrakis(Boc-L-phenylalanyl-3-aminopropyl)-L-α,δ-diaminopentanol* (**9c**): Yield: 54%; C_73_H_110_O_13_N_10_, white foam; ESI MS 1,335 (M+1); ^1^H-NMR (350K, DMSO) δ 1.25-1.35 (m, 36H, C-CH_3,_ Boc), 1.48 (m, 12H, core β, γCH_2_, C-CH_2_-C), 2.34 (m, 6H, core N^δ^-CH_2_), 2.40 (m, 4H, core N^α^-CH_2_), 2.80, 2.97 (dd, *J* = 5.26, 8.55 Hz, 8H, G1 Ar-CH_2_), 3.10 (m, 8H, core C***H***_2_-NH), 3.41 (m, 2H, core C***H***_2_OH), 3.98 (m, 1H, core αCH), 4.20 (m, 4H, G1 αCH), 7.19 (m; 20H, G1 Ar-H). ^13^C-NMR δ 26.5 (γC, δ core C-***C***H_2_-C), 28.5 (βC, αC-***C***H_2_-C), 36.7, 36.8 (α, δ CH_2_NH), 37.7 (G1 Ar-CH_2_), 47.5, 50.9 [core α, δN-(CH_2_)_2_], 53.6 (core δC), 55.5, 55.7 (core and G1 αC), 61.1 (core CH_2_OH), 77.7 [O-***C***-(CH_3_)_3_], 125.2, 125.6, 127.5, 127.2, 128.7, 128.9 (G1 CH_Ar_), 137.6, 138.6 (G1 C_Ar_), 154.3, 154.5 (G1 O-CO-NH), 170.7 (G1 CO-NH).

*N,N',N,N’-tetrakis(Boc-L-phenylalanyl-3-aminopropyl)-L-α,ε-diaminohexanol* (**9d**): Yield: 73.5%; C_74_H_112_O_13_N_10_, white foam; ESI MS 675 (M+2)^2+^, 1,349 (M+1)^+^; ^1^H-NMR (350K, DMSO) δ 1.24-1.36 (m, 36H, C-CH_3_, Boc), 1.43 (m, 14H, core β, γ, δCH_2_, C-CH_2_-C), 2.31 (m, 6H, core N^ε^-CH_2_), 2.42 (m, 4H, core N^α^-CH_2_), 2.79, 2.95 (dd, *J* = 5.27, 8.54 Hz, 8H, G1 Ar-CH_2_), 3.15 (m, 8H, core C***H***_2_-NH), 3.43 (m, 2H, core C***H***_2_OH), 3.99 (m, 1H, core αCH), 4.22 (m, 4H, G1 αCH), 7.20 (m; 20H, G1 Ar-H).^13^C-NMR δ 24.1 (core γC), 26.6 (core δC, ε C-***C***H_2_-C), 28.5 (core βC, αC-***C***H_2_-C), 36.7, 36.8 (core α, ε CH_2_NH), 37.7 (G1 Ar-***C***H_2_-CH), 47.8, 51.0 [core α, εN-(CH_2_)_2_], 53.8 (core εC), 54.9, 55.1 (core and G1 αC), 61.1 (core CH_2_OH), 77.8 [O-***C***-(CH_3_)_3_], 125.2, 125.5, 127.2, 128.7, 129.0 (G1 CH_Ar_), 137.7, 138.7 (G1 C_Ar_), 154.7, 154.7 (G1 O-CO-NH), 170.6 (G1 CO-NH).

#### 3.5.8. Preparation of the dendrimeric compound **10c**

A) **9c** (0.5 g, 0.375 mmol) was dissolved in CH_2_Cl_2_ (5 mL) and TFA (5 mL) was added. The reaction mixture was stirred at room temperature for 3 h. Then the reaction mixture was evaporated *in vacuo*, the residue was dissolved in ethyl acetate (5 mL) and evaporated (three times) and then in diethyl ether (5 mL) and evaporated (twice) – to remove all remaining trifluoroacetic acid. Dark-orange oil (0,4g) was used for the next step without purification.
B) (2-Cl-Z)L-Lys(Boc) (0.68 g, 1.65 mmol) and *N*-hydroxysuccinimide (HOSu, 0.19 g 1.66 mmol) was dissolved in THF (10 mL) followed by addition of DCC (0.34 g, 1.66 mmol). The solution was stirred for 0.5 h and added to the solution of Boc-deprotected **9c** and N(Et)_3_ (1 mL, 7.13 mmol) in DMF (5 mL). The final solution was stirred for 48 h at room temperature, then DCU was filtered off and the solvents was evaporated *in vacuo*. The residue was dissolved in CHCl_3_ (50 mL) and washed with 10% Na_2_CO_3_ and saturated NaCl solution (twice), dried over Na_2_SO_4_ overnight, filtered and evaporated to dryness. The residue was purified by flash chromatography (CHCl_3_:MeOH, 15:1) to give 0.729 g of Boc protected **10c** (77.1%) as a white foam.
C) Boc-**10c** (360 mg, 0.14 mmol) was deprotected according to procedure A). Trifluoroacetate ions were replaced by chlorides by dissolving the oily residue in HCl-saturated ethyl acetate and evaporation *in vacuo* (four times) to give 330 mg of **10c** hexahydrochloride (99%) as a white, hygroscopic foam.

*N,N',N,N’-tetrakis[(N^α^-2-chlorobenzyloxycarbonyl)-L-lysil-L-phenylalanyl-3-aminopropyl]-L-α,δ-diaminopropanol hexahydrochloride* (**10a**): Yield 1.0 g (98%); C_107_H_142_O_17_N_18_Cl_4_·6HCl; pale yellow foam; ESI MS 1,046 (M+2H^+^)^2+^, 1,057 (M+H^+^+Na^+^)^2+^, 2,105 (M+H^+^); ^1^H-NMR (DMSO, 298K) δ 1–2 (bm, 32H, γ,β,δCH_2_ G-1 *Lys*, C-C***H***_2_-C core arms), 2.6-3.7 (4 bm, 37H, C***H***_2_-NH core, εCH_2_ G-1 *Lys* i βCH_2_ core, α, βN-(C***H***_2_)_2_ core, Ar-***C***H_2_-CH *Phe*, C***H***_2_OH & αCH core), 4.1, 4.6 (2 bm, 8H, αCH *Lys* & *Phe,* respectively), 5.16 (4s, 8H, Ar-CH_2_ from *2-Cl-Z*), 7.2 – 7.45 (m; 36H, Ar-H); ^13^C-NMR δ 22.1, 22.3 (γC G-1 *Lys*), 23.4 (α, γC-***C***H_2_-C core), 26.6 (δC G-1 *Lys*), 28.7 (βC core), 30.3, 31.1 (βC G-1 *Lys*), 36.3, 36.5 (α, βCH_2_NH core), 37.7 (Ar-***C***H_2_-CH), 38.3, 38.6 (εCH_2_NH_2_ G-1 *Lys*), 48.8, 49.7 [α, βN-(CH_2_)_2_ core], 52.3 (βC core), 54.2, 55.3 (αC *Phe* and *Lys,* respectively), 58.8 (αC core), 60.3 (CH_2_OH core) 62.7, 62.8 (Ar-***C***H_2_-O), 126.3, 127.2, 128.2, 129.1, 129.3, 129.6, 129.7, 129.8 (CH_Ar_), 132.1, 133.2 (C_ar_-Cl), 134.3, 134.4 (C_ar_-CH_2_*Phe*), 136.9 (C_ar_-CH_2_O), 155.4, 155.5 (O-CO-NH), 171.3, 172.4 (CONH *Phe* and *L-Lys,* respectively); Anal. Calcd for C_107_H_142_O_17_N_18_Cl_4_·6HCl: C, 55.6; H, 6.45; N, 10.9; Cl, 15.33. Found: C, 55.38; H, 6.63; N, 10.58; Cl, 15.02.

*N,N',N,N’-tetrakis[(N^α^-2-chlorobenzyloxycarbonyl)-L-lysil-L-phenylalanyl-3-aminopropyl]-L-α,δ-diaminobutanol hexahydrochloride* (**10b**): Yield 1.01 g (99%); C_108_H_144_O_17_N_18_Cl_4_·6HCl, white hygroscopic foam; ESI MS 1,053 (M+2H^+^)^2+^, 1,064 (M+H^+^+Na^+^)^2+^, 2,105 (M+H^+^); ^1^H-NMR (DMSO, 298K) δ 1 – 2 (bm, 34H, βCH_2_ core i γ,β,δCH_2_ G-1 *Lys*, C-C***H***_2_-C core arms), 2.65-3.75 (3 bm, 37H, C***H***_2_-NH core, εCH_2_ G-1 *Lys* & γCH_2_ core, α, γN-(C***H***_2_)_2_ core, Ar-***C***H_2_-CH *Phe*, C***H***_2_OH and αCH core), 4.15, 4.57 (2 bm, 8H, αCH *Lys* and *Phe,* respectively), 5.11 (4s, 8H, Ar-CH_2_ from *2-Cl-Z*), 7.2–7.45 (m; 36H, Ar-H). ^13^C-NMR δ 22.1, 22.2 (γC G-*L-Lys*), 23.5 (α, δC-***C***H_2_-C core), 26.5 (δC G-1 *Lys*), 28.8 (βC core), 30.2, 31.1 (βC G-1 *Lys*), 36.2, 36.3 (α, γCH_2_NH core), 37.6 (Ar-***C***H_2_-CH), 38.4, 38.5 (εCH_2_NH_2_ G-1 *Lys*), 48.9, 49.7 [α, γN-(CH_2_)_2_ core], 52.0 (γC core), 54.1, 55.1 (αC *Phe* & *Lys,* respectively), 58.4 (αC core), 60.1 (CH_2_OH core) 62.6, 62.8 (Ar-***C***H_2_-O), 126.2, 127.3, 128.1, 129.1, 129.3, 129.5, 129.7, 129.8 (CH_Ar_), 132.2, 133.3 (C_ar_-Cl), 134.2, 134.3 (C_ar_-CH_2_*Phe*), 137.0 (C_ar_-CH_2_O), 155.3, 155.6 (O-CO-NH), 171.2, 172.6 (CONH *Phe* and *Lys,* respectively); Anal. Calcd for C_108_H_144_O_17_N_18_Cl_4*_6HCl: C, 55.74; H, 6.5; N, 10.83; Cl, 15.23. Found: C, 55.5; H, 6.7; N, 10.51; Cl, 14.94.

*N,N',N,N’-tetrakis[(N^α^-2-chlorobenzyloxycarbonyl)-L-lysil-L-phenylalanyl-3-aminopropyl]-L-α,δ-diaminopentanol hexahydrochloride* (**10c**): Yield: 99%; C_109_H_146_O_17_N_18_Cl_4_*6HCl, white hygroscopic foam; ESI MS 1,051 (M-18+2)^2+^, 1,060 (M+2)^2+^, 1,071 (M+1+23)^2+^, 2,119 (M+1)^+^; ^1^H-NMR (298K, DMSO); NMR: δ 1 – 2 (bm, 36H, core γ, βCH_2_, G2 γ,β,δCH_2_, core C-CH_2_-C), 2.7-3.8 (3 bm, 37H, core C***H***_2_-NH, G2 εCH_2_, core δCH_2_, core α, δN-(C***H***_2_)_2_, G1 Ar-C***H***_2_-CH, core C***H***_2_OH and αCH), 4.1, 4.55 (2 bm, 8H, G1 and G2 αCH), 5.15 (m, 8H, G2 Ar-CH_2_), 7.25 (m; 36H, Ar-H), 8.15 (bm, 21H, N-H). ^13^C=NMR δ 22.2, 22.3 (G2 γC), 23.2 (core α, δC-***C***H_2_-C), 23.9 (core γC), 26.4 (G2 δC), 28.4 (core βC), 30.3, 31.0 (G2 βC), 35.9, 36.2 (core α, δ CH_2_NH), 37.7 (G1 Ar-***C***H_2_-CH), 38.3, 38.6 (G2 εCH_2_NH_2_), 48.9, 49.6 [core α, δN-(CH_2_)_2_], 52.1 (core δC), 54.1, 55.0 (G1 and G2 αC), 57.9 (core αC), 60.3 (core CH_2_OH) 62.7, 62.9 (G2 CH_2_Ar), 126.2, 127.3, 128.0, 129.1, 129.4, 129.6, 129.7, 129.7 (CH_Ar_), 132.1, 133.3 (G2 ***C***_ar_-Cl), 134.3, 134.4 (G1 ***C***_ar_-CH_2_), 136.9 (G2 ***C***_ar_-CH_2_), 155.2, 155.7 (G2 O-CO-NH), 171.3, 172.8 (G1 and G2 CO-NH); Anal. Calcd for C_109_H_146_O_17_N_18_Cl_4_·6HCl: C, 55.92; H, 6.54; N, 10.77; Cl, 15.14. Found: C, 55.68; H, 6.65; N, 10.43; Cl, 14.96.

*N,N',N,N’-tetrakis[(N^α^-2-chlorobenzyloxycarbonyl)-L-lysil-L-phenylalanyl-3-aminopropyl]-L-α,ε-diaminohexanol hexahydrochloride* (**10d**): Yield: 99%; C_110_H_148_O_17_N_18_Cl_4_*6HCl, white hygroscopic foam; ESI MS 1,058 (M-18+2)^2+^, 1,067 (M+2)^2+^, 1,078 (M+1+23)^2+^, 2,133 (M+1)^+^; ^1^H-NMR (298K, DMSO) δ 1–2 (bm, 38H, core and G2 γ,β,δCH_2_, core C-CH_2_-C), 2.6-3.8 (3 bm, 37H, core C***H***_2_-NH, core and G2 εCH_2_, core α, εN-(CH_2_)_2_, G1 Ar-***C***H_2_-CH, core C***H***_2_OH, core αCH), 4.0, 4.49 (2 bm, 8H, G1 and G2 αCH), 5.10 (m, 8H, G2 Ar-CH_2_), 7.20 (m; 36H, G1 and G2 Ar-H), 8.11–8.7 (3 bm, 21H, N-H); ^13^C-NMR δ 22.2, 22.3 (G2 γC), 23.0 (core α, εC-***C***H_2_-C), 24.4 (core γC), 25.2 (core δC), 26.3, 26.4 (G2 δC), 28.6 (core βC), 30.0, 31.2 (G2 βC), 36.0, 36.2 (core α, ε CH_2_NH), 37.7 (G1 Ar-***C***H_2_-CH), 38.3, 38.4 (G2 εCH_2_NH_2_), 48.5, 49.8 [core α, εN-(CH_2_)_2_], 51.3 (core εC), 53.7, 54.0, 54.7 (G1 and G2 αC), 57.4 (core αC), 60.4 (core CH_2_OH) 62.8, 62.9 (G2 CH_2_Ar), 126.2, 127.2, 127.9, 129.2, 129.5, 129.6, 129.7 (G1 and G2 CH_Ar_), 132.1, 133.2 (G2 ***C***_ar_-Cl), 134.2, 134.3 (G1 ***C***_ar_-CH_2_), 137.6 (G2 ***C***_ar_-CH_2_), 155.6, 155.8 (G2 O-CO-NH), 171.0, 171.4, 172.7 (G1 and G2 CO-NH); Anal. Calcd for C_110_H_148_O_17_N_18_Cl_4_*6HCl: C, 56.1; H, 6.6; N, 10.7; Cl, 15.05. Found: C, 55.65; H, 6.76; N, 10.35 Cl, 14.91.

## 4. Conclusions

Two series of new low molecular weight amphiphilic peptide dendrimers were efficiently synthesized and characterized. They contain novel core elements - basic *tris*-amino acids **3** and *tetrakis*-amino alcohols **4**. Their application in the synthesis of amphiphilic peptide dendrimers yielded molecules with (+5)/(+6) charge and multiple distribution of cationic and lipophylic groups in the 1st dendrimer generation. These cationic dendrimeric peptides were significantly more potent against Gram(+) and Gram(-) bacteria and fungi from *C. albicans* genus, with single micromolar MICs, than previously designed derivatives built around a Lys(Lys)_2_ core. Interestingly, for the first time high activity of the dendrimeric species against antibiotics resistant MRSA ATCC 43300 and ESBL ATCC BAA-198 pathogens was detected. Both, higher antimicrobial potency and higher hemolytic properties are associated with higher charge and hydrophobicity of the present dendrimers. The high potency and broad range of activity of the new and previously described compounds evidences rationality of searching for simple, economically attractive antimicrobial compounds in the class of low molecular weight peptide dendrimers. 
